# Breaking bad news with reflective AI virtual experience (BRAVE): development and evaluation of a generative AI-based simulation tool

**DOI:** 10.1186/s41077-026-00429-8

**Published:** 2026-03-03

**Authors:** Orit Karnieli-Miller, Zohar Elyoseph

**Affiliations:** 1 Department of Medical Education and Dr. Sol Amsterdam and Dr. David P. Schumann Endowed Chair in Medical Education, Gray Faculty of Medical and Health Sciences, Tel Aviv University, Tel Aviv, 69978 Israel; 2 School of Therapy, Counseling, and Human Development, Faculty of Education, University of Haifa, Haifa, Israel

**Keywords:** Delivering bad news, Breaking bad news, Difficult conversations, AI simulator, Communication skills training, Medical education, Generative AI, Clinical competence, Virtual patient

## Abstract

**Background:**

Breaking bad news (BBN) to patients is complex and emotionally and cognitively demanding. The way bad news is delivered has implications for patients, their significant others, and healthcare professionals. Despite the development of structured BBN protocols, many health professionals report insufficient training and low self-efficacy for conducting these conversations. While best-practice BBN education exist, opportunities for repeated practice with high-quality feedback are limited, representing a critical barrier to scalability and global health equity, particularly in resource-constrained settings. We developed BRAVE (Breaking Bad News with AI Reflective Virtual Experience), a generative AI-based simulator that includes a written BBN encounter with a virtual patient. Unlike prior AI simulators that primarily offered structured summary feedback, BRAVE explicitly combines a prompted, dialogic reflective debrief followed by personalized feedback mapped to the SPwICES protocol.

**Methods:**

A prospective, single arm, pre-post design, which incorporates both quantitative and qualitative measures. Ninety-nine senior medical students completed one BRAVE session following a required BBN course. Pre/post questionnaires assessed self-efficacy, willingness to engage in BBN, and intention to apply the BBN protocol. User experience was rated on a 5-point scale. Quantitative analysis included paired-sample t-tests and descriptive statistics. Qualitative data from open-ended responses were analyzed using immersion/crystallization.

**Results:**

Self-efficacy increased from M = 6.95 to M = 7.62 (*p*<.001), willingness from M = 5.80 to M = 6.51 (*p*<.001), and intention to apply the protocol from M = 8.20 to M = 8.41 (*p*=.005). User experience was positive: interaction quality (M = 4.27), structured feedback (M = 4.19), reflective dialogue (M = 3.91), and interest in future use (M = 3.81). Qualitative analysis showed most students described the simulator as realistic, useful, and safe for practice, emphasizing opportunities to think, reread, and make mistakes with reduced embarrassment; a minority noted limits of the written modality (restricted nonverbal cues and occasional recognition errors), which were addressed during refinement.

**Conclusions:**

A brief text-based GenAI simulation integrating dialogic reflection with protocol-based feedback yielded improvements in learners’ confidence and willingness to conduct BBN and was well-received. BRAVE can complement existing curricula by offering scalable, psychologically safe, and feedback-rich practice; future controlled studies with performance-based outcomes are warranted.

## Introduction

Breaking bad news (BBN) to patients and/or their family members is complex and challenging for healthcare professionals. During these conversations, the news bearer must identify and respond empathically to patients and/or their significant others, choose a tone and pace appropriate to their informational, cultural, emotional, and cognitive needs while remaining hopeful and helpful in guiding toward treatment [1–3]. This is critical as the quality of BBN encounters impacts recipients’ psychological well-being, treatment adherence, news acceptance, coping, and bereavement process; [3–9] as well as professionals’ mental health, burnout, and work satisfaction [10–12]. 

The importance and challenges of managing BBN encounters have led to the understanding of the need for comprehensive BBN training and improved educational pedagogies to support clinical practice [7–9, 11, 13–15]. Thus, structured protocols such as SPIKES or SPwICES were developed [3, 16], which guide professionals through challenging conversations: choosing a private Setting, exploring the patient’s Perception, prior to sharing the news, providing a warning call to help prepare toward the bad news, sharing clear Information with Empathy while ensuring patient understanding through Clarification, and finally, Summarizing and collaboratively developing a Strategy to move forward. Research shows that effective BBN education requires theoretical learning of a gold-standard BBN protocol and experiential training in a safe environment that incorporates reflection as well as individualized feedback [7, 15]. 

However, BBN training faces significant challenges due to limited integration of experiential learning and hands-on practice [7, 14, 17]. Insufficient hands-on practice can be explained by the ethical challenges involved in practicing on real patients; the high cost of simulations with standardized patients (requiring space, time and paying standardized patients), and learners’ embarrassment [3, 7, 18, 19]. An additional challenge is the need for high-quality personalized feedback, which is time-intensive and requires facilitators with expertise in BBN protocols and in providing reflective and constructive feedback [3, 7, 19]. Consequently, most physicians (60%) and medical students (74%) report receiving little or no training [20]. Many professionals report feeling unprepared, with low self-efficacy and high stress, leading to avoidance of BBN or poor communication [11, 20, 21]. This gap highlights a need to develop additional and scalable training methods.

One possible training opportunity is using Generative Artificial Intelligence (GenAI), particularly large language models (LLMs). LLMs can generate contextually sensitive, human-like language based on extensive training data. This capability has begun to be explored in healthcare settings for tasks such as emotion recognition, suicide prevention, and, recently, for teaching basic communication skills such as history taking, showing early signs of effectiveness [22–29]. 

Building on this momentum the current study introduces and evaluates a Breaking Bad News with AI Reflective Virtual Experience (BRAVE) training tool. BRAVE is a GenAI-powered simulator focused on emotionally charged, high-stakes communication. It allows users to engage in a written BBN conversation with a virtual patient and then receive two levels of feedback. In contrast to earlier AI-based tools that focused on automated summaries, BRAVE is deliberately designed to elicit learner reflection through a brief dialogic debrief and then deliver protocol-mapped feedback aligned with SPwICES.

Reflective practice (reflection) is encouraged in medical education [30–35], particularly in simulation practices of teaching communication skills [11, 13, 32, 36–38]. Reflection, as a metacognitive process [39], includes deepening one’s understanding of self, others, and situations; focusing on cognitive, emotional, and behavioral aspects; and connecting between past, present, and future [40]. Although reflection is widely recognized as essential in the feedback process [15, 40–42], acknowledged as important for learning in AI-based studies [18], previously developed AI simulators provided only structured, summary feedback, without explicitly inviting learners’ reflection [27]. Brave was designed to integrate reflective practice.

Thus, the present study has two primary aims. First, to introduce BRAVE, developed as a pedagogical tool for practicing BBN skills. Second, to assess its impact based on the first two levels of the Kirkpatrick model [43], evaluating students *reaction* to BRAVE – i.e., the extent to which they are satisfied with it, find it engaging, and perceive it as relevant to their professional practice; and their *learning*, i.e., its impact on their confidence and competence.

## Methods

### Study design

Consistent with established guidance for pilot studies of novel interventions [44] this study employed a prospective, single arm, pre-post design, which incorporates both quantitative and qualitative measures. This design was used to evaluate students’ reaction (i.e., acceptability and satisfaction with the training), and their learning (i.e., changes in self-efficacy, willingness to deliver bad news, and intention to use the SPwICES BBN protocol) before and after engaging with the BRAVE simulator. The simulation intervention is reported in accordance with the recommended guidelines for simulation-based research by Cheng et al. [45], to ensure clarity, transparency, and replicability.

### Procedure

Brave simulator was integrated as a one-time experiential component within the mandatory BBN course. After learning the SPwICES BBN protocol, practicing it through role-play with feedback, and writing a reflective narrative based on clinical observations, students were invited to use the BRAVE simulator. Immediately before using the simulator participants completed pre-intervention questionnaires. Then, participants accessed the simulator via CalStudio (formerly known as PMFM (PayMeForMYAI); https://calstudio.com/), a no-code AI development platform. This tool is designed to make conversational AI bots accessible to end-users. Participation was individual and asynchronous, with students engaging in the simulation remotely using their own personal computer or mobile device. Each session involved a simulated written BBN dialogue of up to 30 conversational exchanges (20–30 min), followed by an automated reflective and structured feedback component. Students were instructed to complete the full simulation once but were encouraged to revisit and repeat the experience voluntarily for further practice. They subsequently completed post-intervention questionnaires measuring the same variables, along with user experience and satisfaction.

The study received approval from the Tel Aviv University Ethics Committee. Inclusion criteria were senior (final-year) medical students who participated in the full mandatory BBN course during the study period and completed the BRAVE simulation. Students who did not complete the course, did not engage with the simulation, or had incomplete pre- or post-intervention questionnaires were excluded from the analysis. As this was a pilot study embedded within a mandatory course, the sample comprised all eligible students who participated during the study period. No a priori sample size calculation was performed.

### Simulator design

The BRAVE simulator’s design is built on current understanding of healthcare communication education [15, 17]. BRAVE incorporates Experiential Learning Theory (ELT), including the stages of the learning cycle: concrete experience (simulated dialogue), reflective observation (prompted reflective feedback), abstract conceptualization (structured protocol-based feedback), and partial active experimentation (opportunity to clarify and reapply insights) (see intervention design and structure) [46]. It is based on simulation education best practices (e.g., a safe space, authenticity, the importance of prebriefing and debriefing), evidence-based teaching of BBN, including reflective practice and feedback [3, 13, 40, 47]. 

The BRAVE simulation was designed to allow practice and provide feedback, and through that, to enhance students’ self-efficacy in conducting difficult conversations with patients; increase their willingness to engage in bad news delivery; and promote intention to apply the SPwICES communication protocol in clinical settings.

### Prompt engineering and initial testing

The simulator is governed by a single, sophisticated prompt that integrates: Dual personas: “Oron” (patient) with a detailed biography and emotional trajectory (see Table 1); “Dani” (feedback provider) with pedagogical rules and tone (see Table 2). This rich prompt was reinforced with few-shot examples (i.e., a small number of illustrative demonstrations) that provided the model with concrete demonstrations of appropriate dialogue for specific conversational junctures. For example, “Dani” was designed to “acknowledge the challenges and complexity of the task” and “Provide direct and clear feedback, delivered in a respectful and supportive manner.” Furthermore, the prompt contains a detailed, embedded protocol for delivering feedback based on the SPwICES model [3]. This includes precise criteria for evaluating the user’s performance, instructions to use direct quotes for substantiation, and guidelines for maintaining an empathetic and constructive tone. The simulator does not rely on a predefined script; instead, all patient responses are dynamically generated by the LLM in real time, based on user input and dialogue examples (Table 1).

**Table 1:**
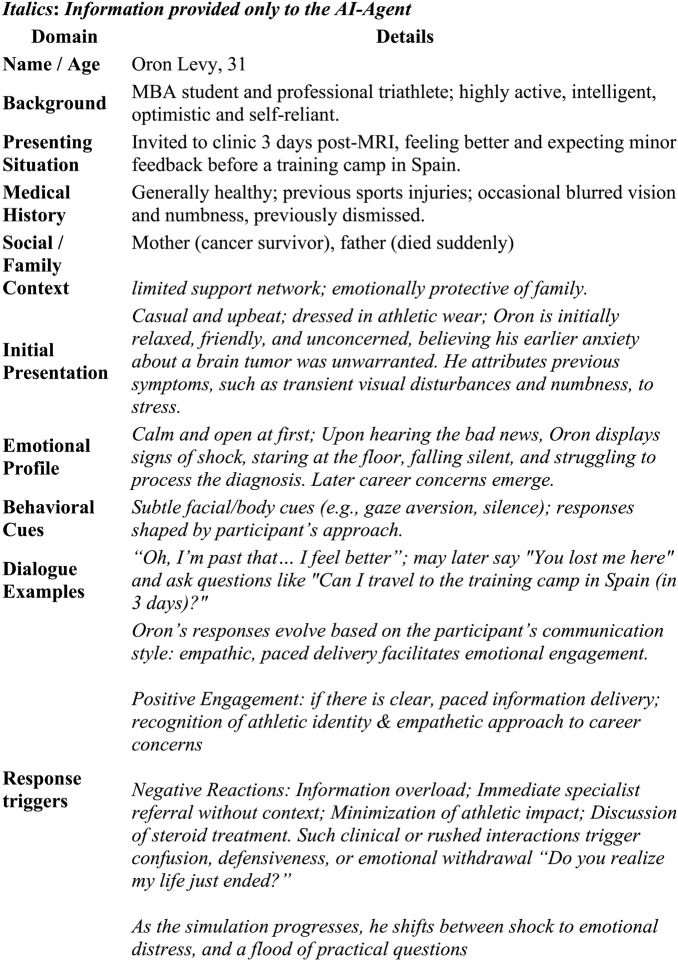
Virtual patient profile - Oron's character in breaking bad news simulator

**Table 2:** Virtual feedback provider profile – “Dani”

Feedback Stage	Primary Focus	Type of Learner Engagement	Example Prompts Used by “Dani”	Pedagogical Rationale
Dialogic Debriefing	Emotional and experiential reflection	Feedback provider invites the learner to share an affective reflection and self-assessment	“What did you feel went well in this conversation?” “What felt challenging for you?”	To surface learners’ emotional responses, normalize difficulty, and establish psychological safety as a foundation for learning. Placing the learner’s experience at the center supports emotional ventilation and prepares learners for deeper reflection and subsequent feedback [47, 48].
	Metacognitive reflection linking emotions, values, and communication choices	Feedback provider invites the learner to elaborate and reflect more deeply	“What do you think influenced how you responded at that moment?” “Why do you think this part felt difficult?”	To help learners connect emotional reactions with underlying assumptions, values, and communication decisions, and to identify learning needs prior to receiving external, protocol-based feedback [39, 40].
Structured Protocol-Based Feedback	Protocol-based analytic feedback grounded in the SPwICES framework	The learner receives structured, evidence-based feedback	“In the Perception stage, you checked the patient’s understanding by saying…” “An alternative phrasing could be…”	To provide clear, concrete, and actionable feedback aligned with the SPwICES protocol. Using direct quotes from the learner’s conversation supports specificity and transfer to future practice. Empathic framing at the outset of written feedback aims to reduce defensiveness and support learner engagement with corrective input [47].
Partial Active Experimentation	Feedback consolidation and application	The learner may choose to engage with an optional dialogic clarification and future-oriented reflection	“What did you take from the feedback?” “What additional example or clarification do you want?”	To support consolidation, clarification, and transfer of feedback into future clinical encounters. This dialogic opportunity allows learners to address misunderstandings, deepen conceptual understanding, and receive tailored guidance aligned with individual learning needs

Furthermore, an interdisciplinary team of three experts, comprising clinical and AI specialists, conducted technical and ethical stress-testing designed to assess the prompt’s robustness and the AI’s consistency. This iterative process involved approximately 30 simulation rounds, including deliberate attempts to derail the AI, “break” its persona, and provoke unethical or clinically inappropriate responses. These tests evaluated the system for potential hallucinations and evaluated the accuracy and appropriateness of the feedback mechanism across a range of simulated user performances (excellent, average, and poor). Following each test round, if needed we refined the prompt to avoid problematic reactions in both the virtual patient “Oron” (e.g., providing irrelevant answers or becoming too aggressive) and the feedback persona “Dani” (e.g., negative tone, critical phrasing or inaccurate identification of communication techniques). This cycle of testing and refinement continued until multiple consecutive trials were completed without critical failures.

### Intervention design and structure

The BRAVE simulator is a GenAI-based educational tool powered by Anthropic’s Claude 3.5 Sonnet model. It was developed by a medical educator who is a communication expert (investing 0.7 FTE) and an educational psychologist who is an AI expert (0.3 FTE). BRAVE’s pedagogical design draws on experiential learning theory (ELT) [46], evidence-based BBN protocol [3], and reflective feedback [40, 47]. The simulation was conducted as a written, text-based communication practice as the text-to-speech and voice-based LLM’s remain technically challenging in low-resource languages [49] such as Hebrew.

**Fig. 1:**
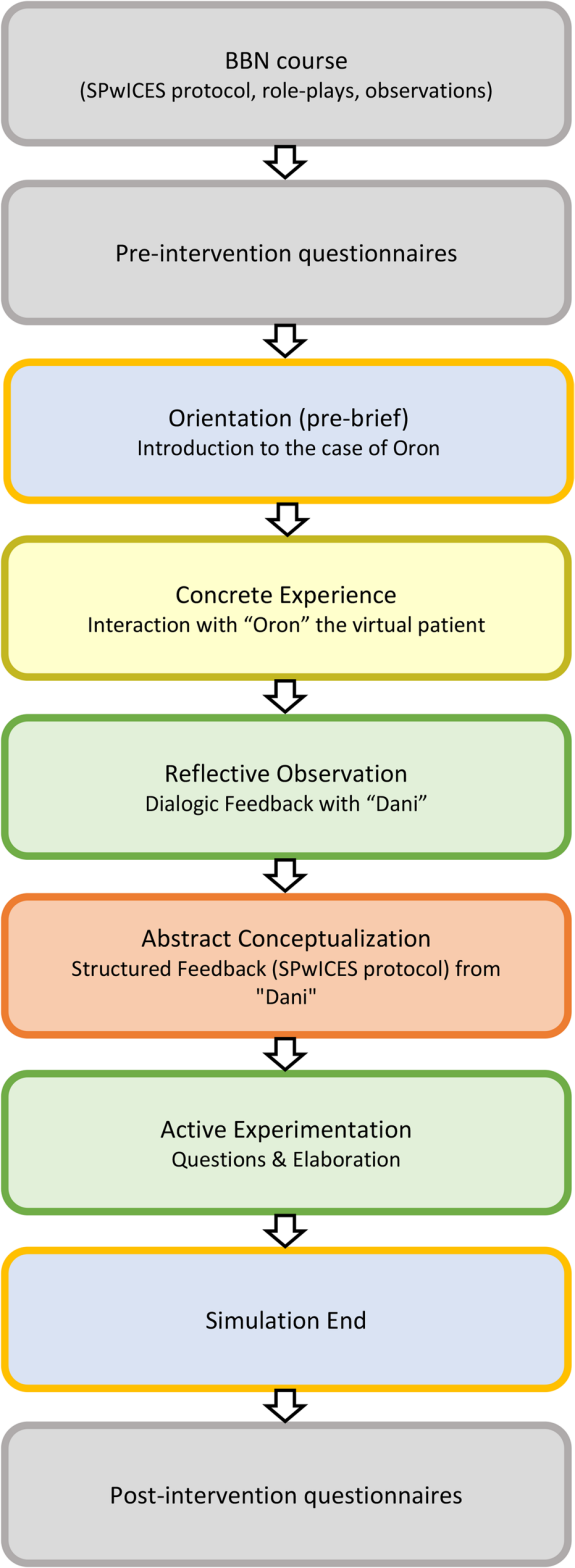
The BRAVE intervention research process

Based on ELT, the simulator comprises five sequential phases (Fig. 1). These phases unfold in a fixed sequence within a single session, with feedback provided immediately after the interaction with the virtual patient.

1. Orientation (pre-brief) – Participants are introduced to the case of Oron Levy, a 31-year-old professional triathlete and MBA student, including his clinical background, who underwent an MRI due to blurred vision and numb feet. Participants are placed in the role of a family physician meeting Oron for the first time tasked with delivering the diagnosis of Multiple Sclerosis.
2. Concrete Experience – Participants interact in a written simulated dialogue with a virtual patient, the AI playing “Oron”. Each of Oron’s response is based on his character and the communication of the student. Oron’s response is limited to 2–3 sentences and includes bracketed nonverbal cues (e.g., “Multiple. what? Wait. isn’t that. isn’t that what makes people end up in wheelchairs?” [Face turns pale, slumps back in chair, stares at the floor].)
3. Reflective Observation – Upon command (“give feedback”), the AI shifts to the persona “Dani,” a communication expert, and initiates a reflective debrief. This reflective dialogue differs from standard AI feedback in that it actively invites the learner to self-evaluate, express challenges, and elaborate on their communication decisions. The AI engages in an empathic, open-ended exchange modeled on evidence-based debriefing techniques, encouraging deeper reflection and metacognitive insight. The reflective dialogue was intentionally limited to seven exchanges. This cap was introduced following iterative expert testing to balance depth of reflection with learner burden, given the tendency of generative AI to prolong open-ended dialogue unless explicitly constrained.
4. Abstract Conceptualization – “Dani” provides structured feedback aligned with the SPwICES protocol [3]. This includes explaination of the goal of each SPwICES component, with examples taken from the student’s conversation illustrating strengths and areas for improvement.
5. Partial Active Experimentation focused on questions, elaboration, or requesting clarification. The AI responds with additional examples or elaborations.

**Fig. 2:**
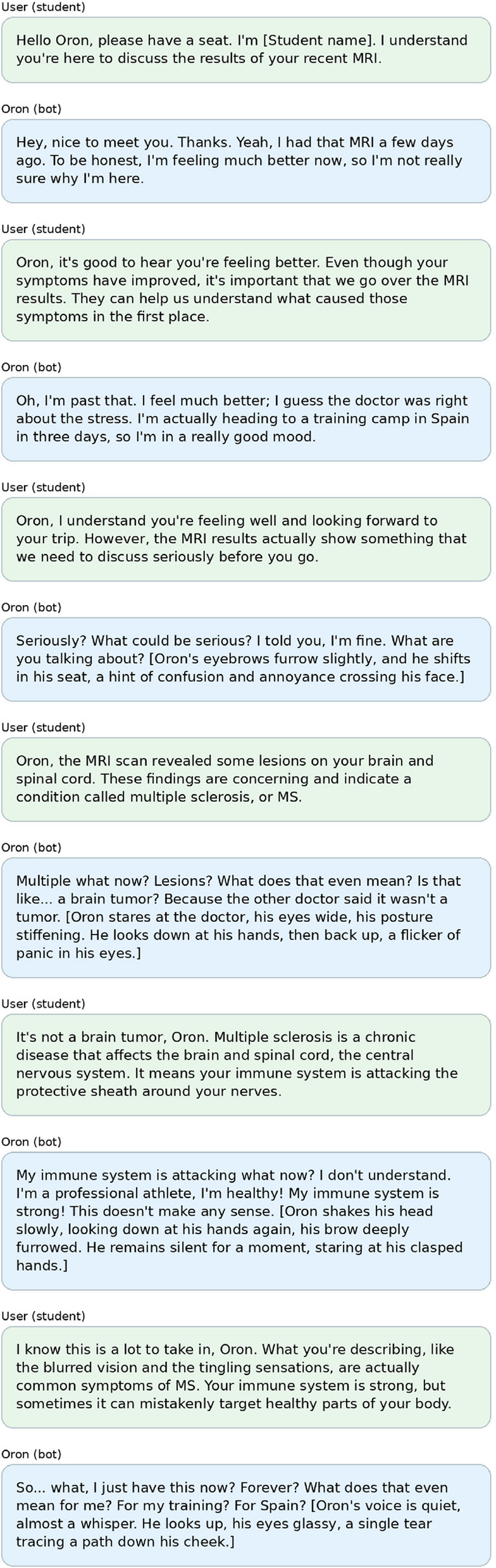
Illustrative excerpt from a trainee’s interaction with the BRAVE simulator, depicting the virtual patient “Oron.”

**Fig. 3:**
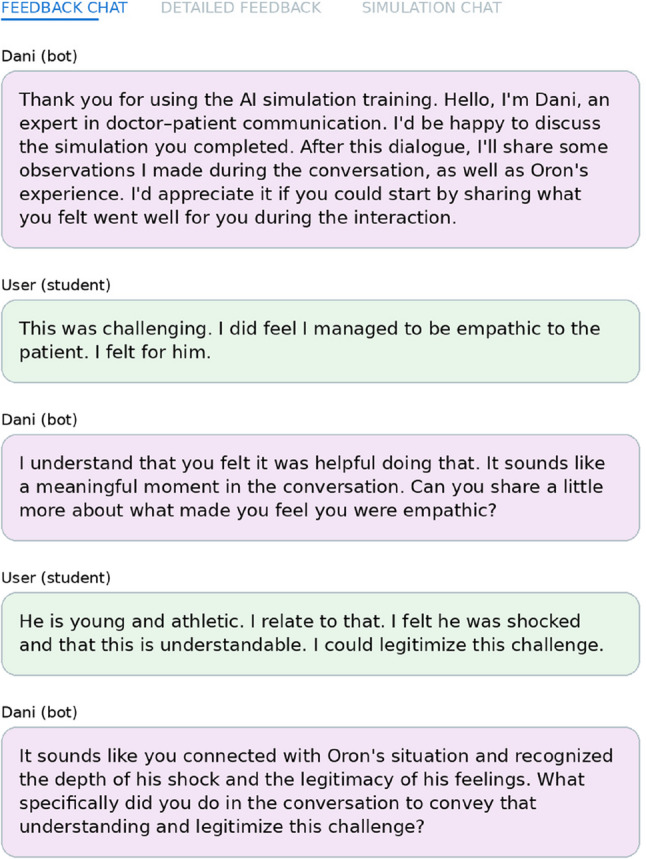
Illustrative excerpt from the trainee’s reflective dialogue with “Dani,” the AI-based communication expert

**Fig. 4:**
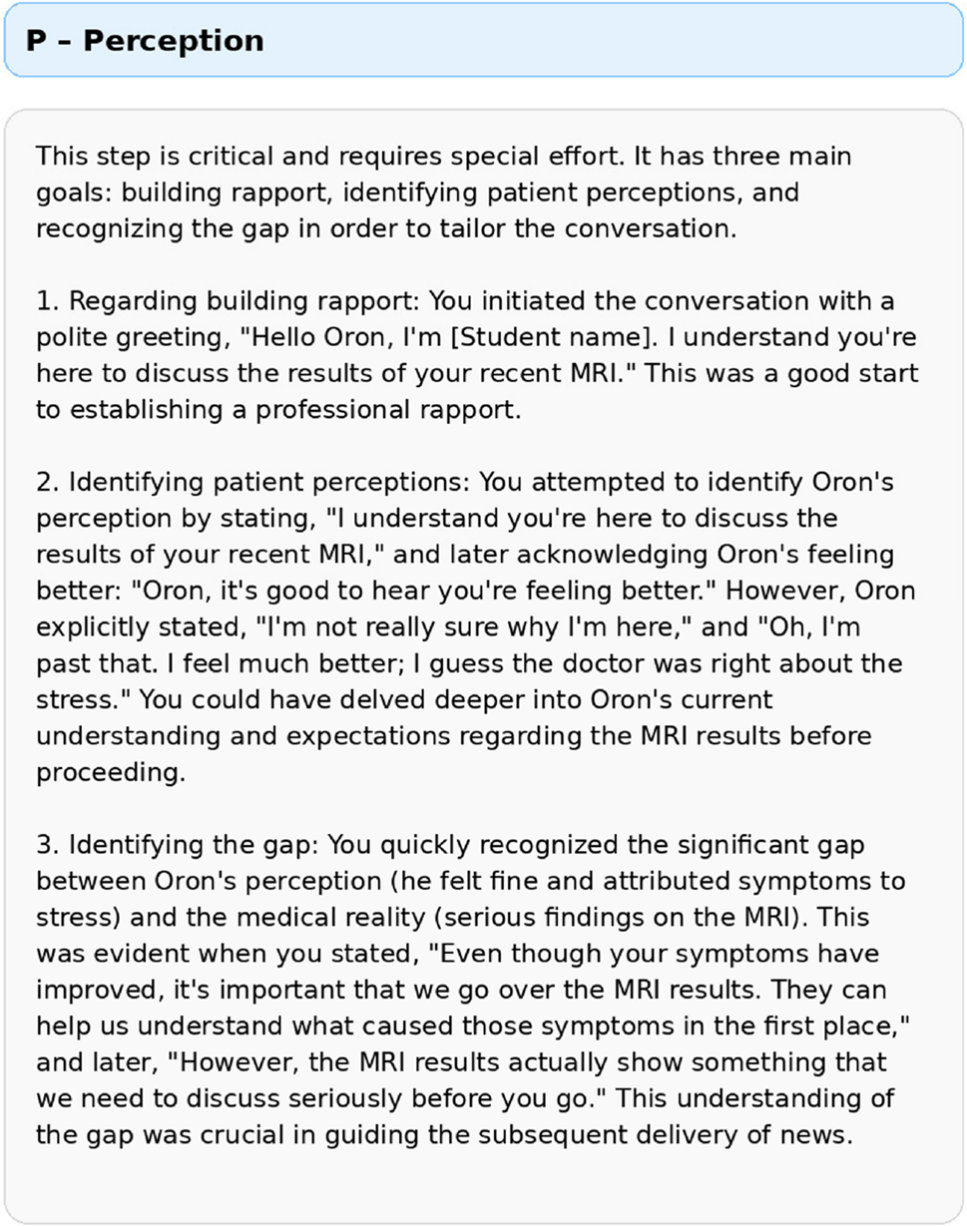
Illustrative excerpt from the structured, protocol-based feedback following the BRAVE simulation, focusing on the P (Perception) component of the SPwICES protocol

Figures 2, 3 and 4 present selected illustrative excerpts from a mock trainee interaction across Phases 2–4 of the BRAVE simulation. The simulator was originally implemented in Hebrew; however, to enhance clarity and accessibility for an international readership, all illustrative examples included in the manuscript are drawn from the English-language version of the BRAVE simulation.

For additional pedagogical evaluation a panel of 12 expert physicians, who specialize in BBN teaching completed the full BRAVE simulation experience (i.e., breaking the news to the patient and receiving reflective and structured feedback). Following this experience they provided qualitative feedback on its educational value, usability, and value prior to implementation. The consensus from this process confirmed the simulator’s readiness for deployment to students.

### Measures

Self-efficacy and willingness were assessed using scales adapted from prior AI-based clinical simulator research [29, 50, 51]. Items were rated on a 10-point Likert scale [1–10], where higher scores represented greater agreement [52]. To examine self-efficacy, participants were asked immediately before using BRAVE three items assessing perceived capabilities, competence, and skills in delivering bad news (pre: α = 0.83; post: α = 0.90), similar to an earlier study [51]. To assess participants’ willingness to engage in BBN encounters, we asked: “If given the opportunity, to what extent would you want to manage conversations in which bad news is delivered to a patient?” To evaluate participants’ perceived understanding of the SPwICES protocol and intent to use it. Two items (understanding + intent) were averaged (pre: α = 0.54; post: α = 0.74).

Post-intervention participants completed the same questions, as well as rated BRAVE components (Oron interaction, reflective and structured feedback, elaboration phase) on a 5-point Likert scale. Because this was a pilot study and we sought to capture learners’ reactions to BRAVE, the post intervention questionnaire also included open-ended questions (e.g., inviting them to share their experience; to compare the BRAVE simulation with traditional role-play).

### Analysis

Quantitative analysis: We conducted paired-sample t-tests to compare pre-post scores; user-experience ratings were summarized descriptively. Qualitative analysis: Open-ended responses were analyzed using the immersion/crystallization approach [53, 54], involving iterative cycles of close reading and discussion to identify, organize themes and reach consensus [13]. 

## Results

Ninety-nine senior medical students completed the simulator. The sample comprised 55 women (55.6%) and 36 men (36.3%), with 8.1% participants preferring not to disclose their gender. Approximately two-thirds (67%) were 26–30 years old, a quarter (24.5%) were 31–35, 6.4% were 22–25, and 2.1% were 36–40.

A paired-samples t-test showed a significant increase in self-efficacy scores from pre-intervention (M = 6.95, SD = 1.18) to post-intervention (M = 7.62, SD = 1.06), t(98) = 9.41, *p*<.001, demonstrating a large effect size (Cohen’s d = 0.95, 95% CI [0.71,1.18]).

Participants demonstrated increased willingness to deliver bad news, with scores rising from pre-intervention (M = 5.80, SD = 2.12) to post-intervention (M = 6.51, SD = 2.08), t(98) = 7.76, *p*<.001, showing a medium-to-large effect size (Cohen’s d = 0.78, 95% CI [0.55, 1.00]).

Participants showed improved implementation intentions, with a significant increase in understanding and intended use from pre-intervention (M = 8.20, SD = 1.37) to post-intervention (M = 8.41, SD = 1.34), t(98) = 2.88, *p*=.005, demonstrating a small effect size (Cohen’s d = 0.29, 95% CI [0.09, 0.49]).

**Fig. 5:**
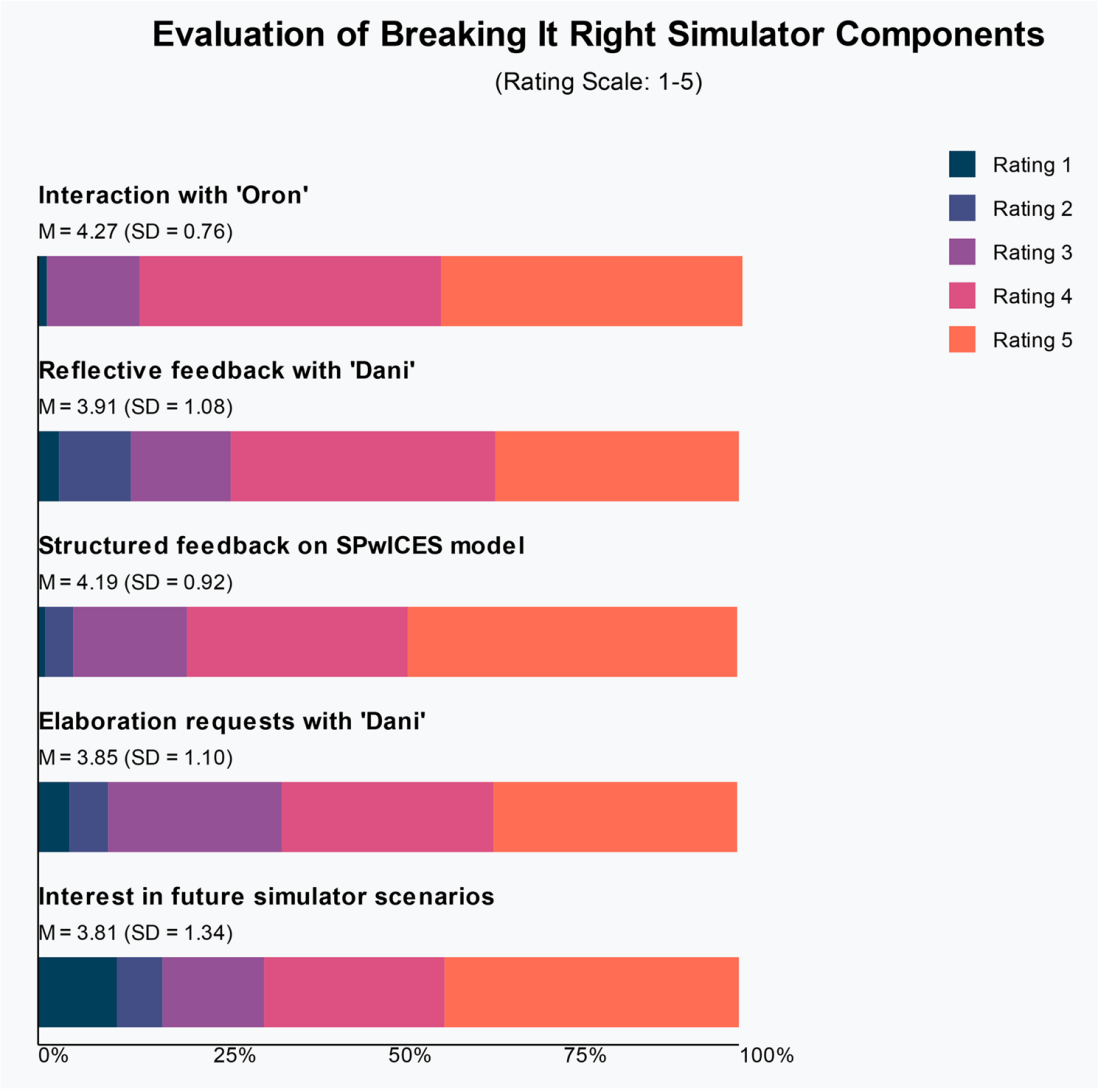
Pre–post comparison of the impact of a *BRAVE* simulation experience on *N* = 99 medical students’ self-efficacy, willingness to deliver bad news, and intention to implement the SPwICES protocol

Participants rated the simulator’s key components on a 5-point scale (see Fig 5). The interaction experience with Oron received the highest ratings (M = 4.27, SD = 0.76), with 85.8% of participants rating it 4 or 5. The structured feedback on the SPwICES protocol also received distinctly positive evaluations (M = 4.19, SD = 0.92). The open dialogue reflective feedback session with Dani was well-received (M = 3.91, SD = 1.08), although 13.3% rated it 1 or 2. The opportunity for feedback elaboration from Dani (M = 3.85, SD = 1.10) received positive ratings from 65.1% of participants. Notably, this feature had a higher nonresponse rate (11%) compared to other components (2%), suggesting that not all participants engaged with this optional feature. Approximately two-thirds (67.7%) indicated great interest in experiencing additional simulator scenarios; however, this component showed the highest variability in responses (M = 3.81, SD = 1.34) (Fig. 5).

### Qualitative analysis of participant responses to the breaking bad news simulator

Qualitative analysis identified four themes. First, most students (94%) described the simulator as positive, realistic, and useful, highlighting practice opportunities and structured, accurate feedback. Second, students reported deeper learning and reflection, with clearer understanding and organization of the BBN protocol. Third, the written, self-paced format created a safe space, providing time to think, reread, and make mistakes, and reducing embarrassment. Finally, a minority noted limitations of the written modality (reduced nonverbal communication and occasional recognition errors), some of which were subsequently resolved.

#### “Very cool, creative, and educational” Students’ perceptions of the learning process and content

Most (94%) students had a *positive learning experience*, highlighting practice, reflection, and helpful feedback. Many students mentioned that they “loved” the simulator. Positive comments included “A great simulation, feels really realistic, except it’s only written”; “Very cool!” a “Great experience; I’d love to practice more.” Many indicated the realistic experience: “Hard to believe it’s not a real person because it feels very professional and realistic,” with a “conversation and responses that felt very *real and not artificial*.” Participants felt it was: “Great, helps clarify what to focus on in our future professional practice.” They identified its *educational value*: “Very cool, creative, and educational. The practical examples at the end were very relevant. I even saved them for later.”

#### “Mostly made me think” Opportunity to enhance reflection and learning

Students appreciated this tool as an *opportunity to enhance reflection and learning*: “It definitely taught us the BBN protocol in a more engaging and varied way. It raised some interesting points and mostly made me think”; “…really helps organize your thoughts at your own pace.” They acknowledged that “a lot of information was given, and it was very systematic.” They found the simulator *helpful in clarifying the BBN protocol*: “… good and provides feedback clearly based on the protocol.” “…does a good job of emphasizing what to focus on according to the BBN protocol.” They appreicated that “Dani’s (structured) feedback touched on the right points and read my [their] strengths and weaknesses accurately.”

#### “Allowed me to practice in a different way” – Benefits and limitations of the simulator’s format and interaction style

When asked to compare the simulator to role-play, students reported that it “felt *similar to the role-play*,” with the “conversation bringing up similar difficulties.” Some stressed that it *retained the benefits of role-play without its drawbacks*: “Like role-play, it allowed practice of the SPwICES protocol. Unlike in role-play, no one was watching me, so I didn’t feel like I was being judged” and felt “less embarrassed.” Others highlighted the freedom it offered: “Allowed me to practice in a different way, just me with myself, so it was easier to make mistakes,” providing “the freedom to say things and express myself in ways I wouldn’t necessarily feel comfortable with in front of a group.” However, a few mentioned the *benefit of nonverbal communication in real-life role-play*, and one said that the entire process was useless and that all other course components were preferable.

Some students highlighted *positive aspects of the written format*, such as: “Having time to think about each response during the conversation.” Providing space to practice more easily: “The setting felt more comfortable, and the ability to go back and reread what I wrote was very helpful”; “Time to think without pressure…helped feel more confident in addressing the illness.” All this led to deeper understanding: “I could reread what I had written and understand why the patient responded the way they did.” Moreover, the format “helped me remember the [BBN protocol] structure and practice using it, identify unexpected responses, and gave me time to plan what to say,” time to “think more deeply about what I was writing and be more precise,” “ to organize my thoughts at ease,” and “feel more in control of the situation.”

A few students raised *concerns about the written format*. “The fact that it’s written feels a bit strange and maybe not fully representative.” Some noted *difficulty in expressing nonverbal communication*: “Talking to a computer makes it harder to express real empathy, even physically, like through silence, tone of voice, hand gestures, and body language.”

Other challenges were related to inaccuracies: “The simulator addresses the doctor only as male. Please fix this and be mindful.” (This issue was resolved.) Another student noted: “It was nice, but the feedback complimented a sentence that the patient said, not me.” Others observed: “The simulator treated some things that were said in the conversation as if they hadn’t been said; maybe because they weren’t phrased exactly as the system expects.” Prompt modifications and a shift to Gemini 2.5 resolved these issues.

## Discussion

This pilot study evaluated BRAVE, a GenAI-driven simulator that allows practice followed by two elements: a brief dialogic reflective debrief and personalized feedback mapped to a protocol (SPwICES). Even a short, text-based session produced improvements in learners’ self-efficacy, willingness to engage in BBN, and intention to apply the protocol, alongside high ratings for interaction quality and feedback components. These results are encouraging, given the well-documented challenges facing health professionals when conducting these emotionally complex conversations.

Furthermore, the simulator’s architecture supports the experiential learning cycle [46]. The BRAVE simulator engages users in Concrete Experience (through an emotionally charged, interactive scenario at a self-selected time and pace), Reflective Observation (enabled by the slowed-down written format [a surprising finding] and through the reflective feedback questions), Abstract Conceptualization (in the structured, personalized feedback that includes positive reinforcement, explanation, and concrete examples), and partial Active Experimentation (via the opportunity to ask for elaboration in a safe environment). A key strength of the GenAI simulator is its ability to provide a psychologically safe, nonjudgmental environment where learners can test communication strategies without observers, time constraints, or risk of harm. This space for reflection and refinement is particularly valuable for novices. To allow the entire ELT cycle, additional opportunities for practice are needed.

The few studies that have explored GenAI-based simulations emphasized their potential to provide learners with an immersive and authentic sense of professional interaction, allowing them to apply communication skills in a dynamic, unscripted dialogue. This effect has been demonstrated in voice recognition-based AI interactions [18] and was evident in our study using a text-based interface. Participants frequently described the simulation and the virtual character as realistic and human-like. This perceived authenticity may be explained by the advanced capabilities of LLMs to generate coherent, context-sensitive responses based on learner input. In our study, the GenAI agent was given the same background information typically provided to standardized patients, enabling a response in a contextually appropriate manner while adapting to the learner’s communication. The GenAI system generated its replies autonomously within the conversational context. These features may support the illusion of spontaneity and emotional responsiveness. As authenticity is a core element of simulation-based learning [55] GenAI’s ability to approximate human dialogue, even in the absence of visual or vocal nonverbal cues, offers a compelling pedagogical advantage.

While this format supports early-stage skill development it is not intended to replace face-to-face, real-time verbal training. As recommended in simulation-based education [56], it should be thoughtfully integrated into a sequenced learning design that includes theoretical instruction, role-play, narrative reflection, observation, and feedback [7]. In this study, the GenAI simulation was implemented as an additional learning modality, after students had engaged in all the above preparatory components, allowing them to apply prior learning in a novel modality, and before practicing again in a face-to-face standardized simulation. Their relatively high baseline scores may reflect this scaffolding, while also indicating room for improvement and needed training.

Even though novice learners often require additional time to practice, reflect, and carefully plan their responses in complex BBN clinical interactions, extended learning is often difficult to support in traditional real-life simulations (where time with standardized patients and facilitators is limited, and when observers may disengage due to lack of involvement). In contrast, GenAI-based simulations offer a flexible and learner-paced environment. The qualitative findings indicated that even though the written format was used because of limitations of LLM’s in low-resource languages [49] the written format itself created a space to pause, reconsider one’s communication strategy, and revise responses before submitting; opportunities that are rarely available in live verbal exchanges. This asynchronous interaction allows learners to process emotions and cognitive challenges at their own pace, enhancing their sense of control and clarity. Future studies should explore the transferability of learning through BRAVE, due to its written format, to standardized simulations and to real-life face-to-face encounters.

Participants perceived all feedback components as relevant and valuable. Although self-reflection is widely recognized as essential in the feedback process and crucial for developing communication skills [40, 42], and despite other AI-based studies emphasizing the importance of reflection in learning [18], previously developed AI simulators typically provided only structured, summary feedback, in which the AI directly shares its evaluation with the student, without explicitly inviting learner reflection. In contrast, in the current study, self-reflection was intentionally integrated at the beginning and the end of the feedback session. Initially, students were explicitly encouraged to reflect on their performance through open-ended questions, prompting them to identify aspects that went well and areas for improvement. This introductory self-reflection may have, similary to face-to-face feedback, played a critical role in building rapport with Dani, the AI-based feedback provider, designed to respond empathically to challenges raised by the learners. The depth and length of this reflective piece were monitored (limited to 7 utterances), as our BBN experts initially felt it was exhausting. Following this reflective phase, “Dani” delivered structured feedback based on the SPwICES protocol, allowing students to compare their self-evaluation with feedback from a virtual “communication expert.”

The study has several limitations. First, this was a prospective, single arm, pre-post design, pilot study without a comparison group design and did not include an alternative intervention unrelated to the simulator. Additional research using randomized controlled designs is needed to compare BRAVE with other instructional methods and establish its effectiveness. Second, the questionnaires used in this study were partially adapted from prior research and demonstrated acceptable internal consistency; however, full psychometric validation was not conducted. Future studies should include validation of these measures. Third, we did not include objective behavioral assessments to evaluate participants’ actual communication performance (e.g., direct observation or performance-based ratings). As a result, we cannot determine whether the self-reported improvements observed after the intervention translated into meaningful changes in clinical behavior (levels 3–4 in the Kirkpatrick model). Future studies incorporating observational assessments or patient feedback are recommended to validate whether gains in perceived competence are reflected in real-world communication outcomes. Fourth, the study focused on one virtual patient scenario, featuring a character with a specific personality (high intellectual level and pleasant demeanor), and only one type of illness. We are working on diverse patient characters, from different gender identities and biological sex, varied emotional, cultural, and cognitive profiles, as well as a range of medical diagnoses, to reflect real-world variability and challenges. Fifth, the predefined limit on the number of reflective dialogue exchanges reflects an early-stage design decision informed by expert testing rather than empirical optimization. Future studies may examine how varying the depth and duration of AI-facilitated reflective dialogue influences learning outcomes. Six, although the written communication format was perceived as authentic and engaging for learning, the absence of crucial nonverbal communication channels, such as facial expressions, tone of voice, and body language, limits the realism and comprehensiveness of the empathy conveyed. It may also limit the ability to transfer these skills to real life face-to-face conversations. Future studies should explore this transference. Seven, as some students may have chosen to practice with the simulator more than once, differences in levels of engagement and learning may exist, highlighting the need for future studies that systematically examine the impact of repeated exposure and number of practice experiences. Eight, while these initial findings are encouraging, future studies should employ comparative and longitudinal designs, with larger and more diverse samples, to better understand the simulator’s long-term effects and its impact across learner subgroups with varying baseline communication proficiency. Finally, given the evolving nature of LLMs, when developing such educational intervention maintaining consistency and empathic quality requires ongoing human oversight. In BRAVE, this is addressed through structured prompting, periodic review of interactions, and integration of the simulator within a supervised educational framework that encourages learner feedback on problematic responses. These require faculty expertise and time. To address technical sustainability and ‘model drift,’ we transitioned from Claude 3.5 Sonnet to Gemini 2.5 Pro, utilizing its extended context window to minimize hallucinations and ensure accurate feedback integration over long interactions. Future iterations will further enhance stability by evolving from a single-prompt architecture to a multi-agent system, assigning distinct roles (e.g., ‘Patient’ vs. ‘Educator’) to specialized agents.

## Conclusions and implications

This pilot study demonstrates the positive impact of an AI-based simulator on medical students’ self-efficacy, willingness to engage in BBN, and intention to apply the SPwICES protocol. The results highlight BRAVE’s potential as a valuable educational tool. While well-received, the simulator is not intended to replace human educators or in-person simulation training, but to complement existing educational approaches by offering another psychologically safe, nonjudgmental space that allows learners to practice difficult conversations on their own time with personalized, reflective and structured feedback. Furthermore, the platform’s scalability and adaptability make it well-suited for teaching foundational communication skills across various health professions, such as nursing, genetics, and veterinary medicine, in which delivering difficult information is critical.

## Data Availability

The datasets generated and/or analysed during the current study are available from the corresponding author.
